# Subseismic to Seismic Slip in Smectite Clay Nanofoliation

**DOI:** 10.1029/2019JB017364

**Published:** 2019-07-29

**Authors:** S. Aretusini, O. Plümper, E. Spagnuolo, G. Di Toro

**Affiliations:** ^1^ Sezione di Tettonofisica e Sismologia Istituto Nazionale di Geofisica e Vulcanologia Rome Italy; ^2^ Department of Earth Sciences Utrecht University Utrecht The Netherlands; ^3^ Dipartimento di Geoscienze Università degli Studi di Padova Padua Italy

**Keywords:** earthquake, microstructure, deformation processes, high velocity friction

## Abstract

Smectite clays are the main constituent of slipping zones found in subduction zone faults at shallow depth (e.g., <1‐km depth in the Japan Trench) and in the decollements of large landslides (e.g., 1963 landslide, Vajont, Italy). Therefore, deformation processes in smectite clays may control the mechanical behavior from slow creep to fast accelerations and slip during earthquakes and landslides. Here, we use (1) laboratory experiments to investigate the mechanical behavior of partly water‐saturated smectite‐rich gouges sheared from subseismic to seismic slip rates *V* and (2) nanoscale microscopy to study the gouge fabric. At all slip rates, deformation localizes in volumes of the gouge layer that contain a “nanofoliation” consisting of anastomosing smectite crystals. “Seismic” nanofoliations produced at *V* = 0.01, 0.1, and 1.3 m/s are similar to “subseismic” nanofoliations obtained at *V* = 10^−5^ m/s. This similarity suggests that frictional slip along water‐lubricated smectite grain boundaries and basal planes may occur from subseismic to seismic slip rates in natural smectite‐rich faults. Thus, if water is available along smectite grain boundaries and basal planes, nanofoliations can develop from slow to fast slip rates. Still, when nanofoliations are found highly localized in a volume, they can be diagnostic of slip that occurred at rates equal or larger than 0.01 m/s. In such a case, they could be markers of past seismic events when found in natural fault rocks.

## Key Points

- Nanofoliations develop at subseismic to seismic slip rates in partly water saturated smectite fault gouges
- Nanofoliations may deform by frictional slip in smectite water‐lubricated basal planes and grain boundaries
- Strain localized nanofoliations can be diagnostic of past slip events at rates larger than 0.01 m/s in natural fault rocks

## 1 Introduction

Scientific drilling evidenced that smectite clay minerals typically constitute the cores of the shallow sections of mature crustal faults (e.g., the creeping section of the San Andreas Fault; Carpenter et al., 2011) and subduction zone megathrust faults (e.g., the Japan Trench megathrust; Kameda et al., 2015). Moreover, smectites often constitute the slipping zone of landslides *decollements* (Hendron & Patton, 1987; Nakamura et al., 2010). In particular, in subduction zone megathrust faults, smectite clay minerals may control the frictional behavior of natural faults at shallow depths (<5–10 km), as smectite at 120–150 °C reacts into interstratified illite‐smectite and then illite (Schleicher et al., 2015). In this shallow depth interval a range of slip events occur, including aseismic creep, episodic slow slip events, and propagation of seismic ruptures nucleating at larger depths, as happened during the Tohoku‐Oki *M*_*w*_ 9.0 earthquake (Chester et al., 2013). Due to the importance of smectite, natural and “analog” fault gouges were intensively studied at both subseismic slip rates *V* = 10^−7^–10^−4^ m/s (Ikari et al., 2009; Morrow et al., 2017; Saffer & Marone, 2003; Wojatschke et al., 2016) and seismic slip rates *V* = 10^−4^–10 m/s (Bullock et al., 2015; Ferri et al., 2011; French et al., 2014; Ujiie et al., 2011). At subseismic slip rates, in saturated smectite‐rich gouge layers friction coefficient was 0.1–0.15 at slip rates that allowed reequilibration of the pore fluid pressures (i.e., typically below 0.5 μm/s; Faulkner et al., 2018; Morrow et al., 2017). At subseismic slip rates deformation was localized within a foliation subparallel to Y‐ and R1‐type microshear zones (Logan et al., 1992) crosscutting a matrix containing a foliated “S‐shaped fabric” (Wojatschke et al., 2016). Instead, when sheared at 1 m/s (seismic slip rates), in partly saturated smectite‐rich gouge (i) the (apparent) friction coefficient was between 0.1 and 0.05 (e.g., Faulkner et al., 2011; Remitti et al., 2015; Ujiie et al., 2013, 2011) and (ii) deformation was localized in 200‐ to 500‐μm‐thick microfoliations (French et al., 2014), which were also observed at the nanoscale but without a precise control on the sampling position (Ujiie et al., 2011). Due to the low hydraulic conductivity typical of smectite clays, shear‐enhanced compaction is expected to produce a transient (“mechanical”) pore pressure increase leading to a decrease of the apparent friction coefficient for *V* = 0.5–5 μm/s (Faulkner et al., 2018). The apparent friction coefficient should further decrease at seismic slip rates (*V* > 10^−4^ m/s) for the additional transient fluid pressure increase due to the temperature increase by frictional heating that induces the thermal pressurization of pore fluids (Faulkner et al., 2011; Rice, 2006; Veveakis et al., 2007) and the thermochemical pressurization of water expelled from the basal planes (Ferri et al., 2011).

Most of the previous experimental studies focused on the determination of the frictional strength of smectite‐rich gouges and on the investigation of the transient mechanical and thermal pore fluid pressurization processes. However, only few studies exploited nanoscale observations to investigate the deformation mechanisms that control frictional strength in clay‐rich gouges at subseismic slip rates and none of these studies, at least to our knowledge, investigated fault materials produced at seismic slip rates. To determine the nanoscale deformation mechanisms operating from subseismic to seismic slip rates in partly saturated smectite‐rich gouges, we use an integrated approach that included experimental rock mechanics and multiscale electron microscopy. To overcome limitations in site‐specific sampling of nanostructures associated to smectite deformation in highly localized shear zones, we employed focused ion beam scanning electron microscopy (FIB‐SEM)‐assisted sample preparation. Based on novel high‐resolution structural and chemical analysis obtained from analytical transmission electron microscopy (TEM) we show that a very similar nanofoliation microstructure, possibly produced by frictional sliding along water‐lubricated smectite basal planes and grain boundaries and rotation of grains, is found in the gouges sheared from subseismic to seismic slip rates.

## 2 Materials and Methods

We deformed experimentally at subseismic and seismic slip rates a granular material (gouge) made of 70 wt.% Ca‐montmorillonite (smectite clay) and 30 wt.% opal‐ct (Chipera & Bish, 2001). The gouge had a median grain size of 7 μm, measured with the multiwavelength extinction profile method (Text S1 in the supporting information; Detloff et al., 2011). This gouge mixture was selected because smectite‐opal association occurs as alteration of tephra layers within the subducting sediments at shallow depth (Vrolijk, 1990) or of volcanic rocks on the subsurface and, therefore, it reproduces the mineral assemblage found in the smectite‐rich fault core of the Japan Trench megathrust (Kameda et al., 2015), or in the slipping zone of large landslides (e.g., Vajont, Italy, Hendron & Patton, 1987). Moreover, this mixture of smectite and opal was investigated in depth by means of crystallographic, mineralogical, and experimental rock‐deformation studies (Aretusini et al., 2017; Chipera & Bish, 2001; Viani et al., 2002).

**Figure 1:**
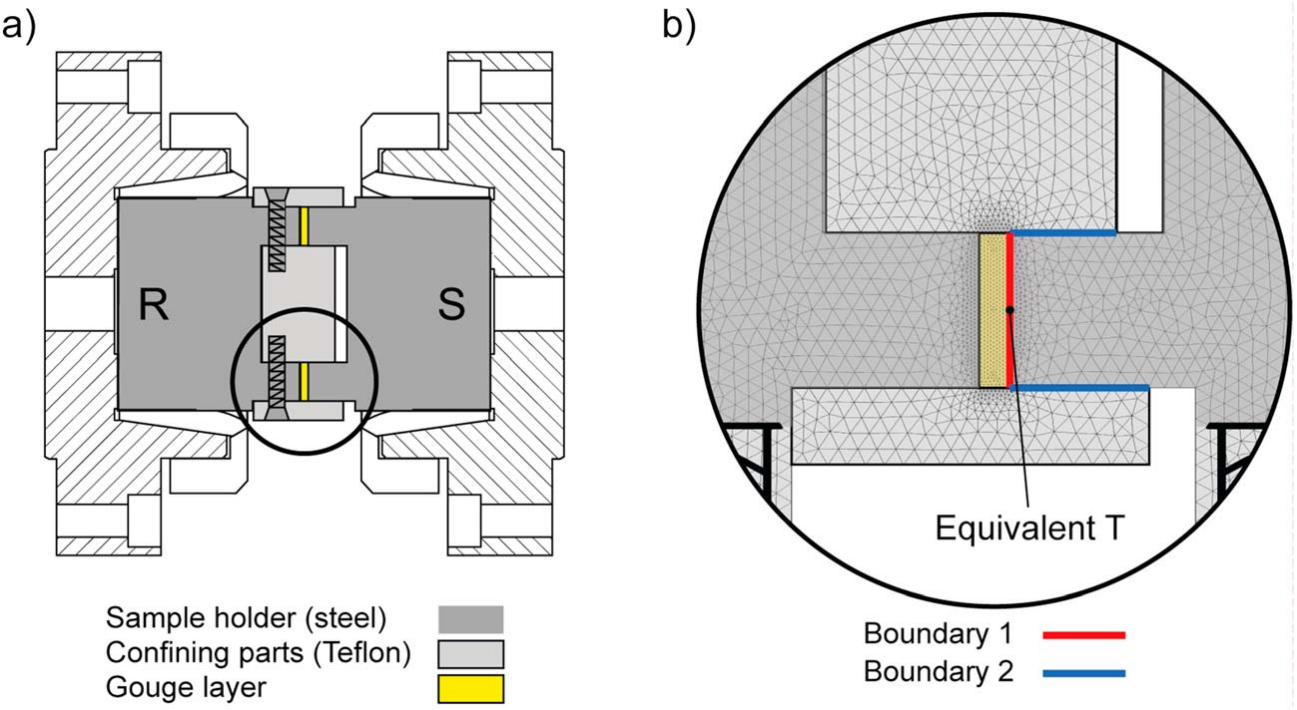
Experimental and numerical modeling methods. (a) Sample holder in radial section (R and S indicate rotating or rotary and static or stationary parts). (b) Geometry with overlaid mesh of the thermal model. The position at which equivalent temperature is calculated is indicated at the edge of the gouge layer.

Ten experiments were performed at subseismic (*V* = 10^−5^ m/s) and seismic (*V* = 0.01, 0.1, and 1.3 m/s) slip rates under a constant normal stress of 5 MPa using the rotary shear machine SHIVA (see the full description of the machine, acquisition system, and calibration of the several devices installed in Di Toro et al., 2010; Niemeijer et al., 2011). The imposed acceleration in all experiments was 6.4 m/s^2^. Two‐millimeters thick, ring‐shaped gouge layers (50‐mm external diameter, 30‐mm internal diameter) were placed between two hollow steel cylinders and confined using Teflon inner and outer rings (Figure 1a). Prior to the experiment, 0.5 mL of deionized water was added to the gouge layer to achieve partly saturated conditions. The gouge layers were sheared for a total displacement of 0.1 m (at all slip rates) and 3 m (only at seismic slip rates). An experiment (s1198) performed at 1.3 m/s under room humidity conditions is reported from Aretusini et al. (2017) for comparison with the partly saturated experiments. During the experiments we measured (i) the shear stress *τ*, calculated from the torque measured on the stationary column (Shimamoto & Tsutsumi, 1994) from which we subtracted the torque exerted by the sample holder to account for sliding between rotating Teflon‐confining parts and stationary steel sample holder (Sawai et al., 2012), (ii) the normal stress *σ*_*n*_, (iii) the apparent friction coefficient *μ′*, calculated as *τ/σ*_*n*_, which differs from the friction coefficient *μ*, calculated as *τ/(σ*_*n*_ *− P*_*p*_ *)* because pore pressure *P*_*p*_ was not monitored nor controlled, and (iv) axial shortening associated to dilatancy and compaction in the slipping zone. Axial shortening was normalized with respect to the initial thickness of the gouge layer in the data set presented in this study.

The temperature evolution within the gouge layer was modeled with a 2‐D axisymmetric finite element thermal conduction model using COMSOL Multiphysics software. The geometry of the sample assemblage plus gouge layer was reproduced in the numerical model (Figure 1b). The dimensions of the mesh elements were reduced in the gouge layer zone and the thermal properties ware attribute to each domain: AISI304 steel for the sample holder, Teflon for the solid confining medium and smectite plus opal for the gouge layer (Table 1). Heat sources *Q*1 and *Q*2 were calculated as variable in time *t* and radius *r* of the model as follows:

(1) $$Q1\left(t,r\right)=\tau \left(t\right)\;V\left(r,t\right)$$

(2) $$Q2\left(t,r\right)=\tau_{T-S}\left(t\right)\;V\left(r,t\right),$$

respectively, with τ being the shear stress of the gouge layer (corrected as described above), *τ*_T‐S_ the shear resistance between the rotating Teflon parts and the stationary steel holder (calculated as in Kitajima et al., 2010), *V* the slip rate. We assumed that 100% of frictional power was converted into heat during slip. The initial model temperature was measured before the experiments using a K‐thermocouple (*T*_0_, Table 2). In the modeled data reported in this study we considered the output temperature (i.e., equivalent temperature) modeled at a nodal point within the gouge layer at the equivalent radius, located approximately halfway across the internal and external radius of the sample holder (Figure 1b and Table 2).

**Table 1:** Thermal Properties of the Thermal Model Domains

Model domain	Thermal conductivity	Heat capacity	Density	Maximum mesh size
	(W/(m·K))	(J/(kg·K))	(kg/m^3^)	(mm)
Gouge (room humidity)^a^	1.2	813	1,267	0.25
Gouge (partly saturated)^b^	1.1	1,269	1,231	0.25
Steel (AISI304)^c^	16	500	8,000	1.25
Teflon^c^	0.24	1,050	2,200	1.25

a Thermal properties from Plotze et al. (2007).

b Weighted average of smectite and water thermal properties (estimated porosity of 35%).

c Thermal properties from Comsol Multiphysics material library.

**Table 2:** Summary of the Experimental Conditions, Microstructural and Modeling Results

	Experiment					Model		
Name	Slip rate	Slip	Duration	Thickness deformed domain	Maximum strain rate	Initial *T*	Maximum equivalent *T*	*T* increase
	(m/s)	(m)	(s)	(μm)	(1/s)	(°C)	(°C)	(°C)
s1529	0.00001	0.1	11,480	300	0.033	29.2	29.25	0.05
s1333	0.01	0.1	10	n.a.^b^	n.a.^b^	19.0	21.6	2.6
s1338	0.01	0.1	10	300	33	24.5	28.2	3.7
s1334	0.1	0.1	1	250	400	19.5	38.6	19.1
s1335	1.3	0.1	0.08	150	8,667	19.9	42.8	22.9
s1253	0.01	3	300	1,400	7	24.3	44.3	20.0
s1252	0.1	3	30	1,400	71	22.9	103.3	80.4
s1251	1.3	3	2.5	150	8,667	26.1	63.6	37.5
s1166^a^	1.3	3	2.5	n.a.^b^	n.a.^b^	29.1	291.2	262.1
s1198^a^	1.3	3	2.5	1,000	1,300	n.a.^b^	n.a.^b^	n.a.^b^

a Experiments performed under room humidity conditions (Aretusini et al., 2017).

b Data not available due to incomplete sampling of microstructure or missing temperature measurement.

**Figure 2:**
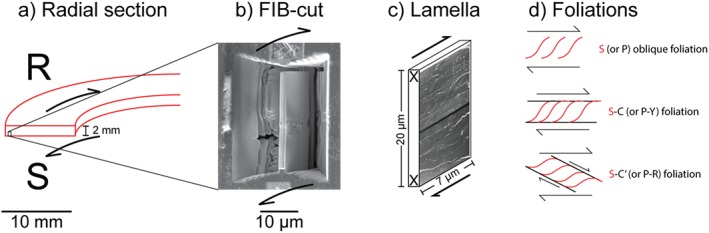
Microanalytical methods. (a) Orientation of sample sectioning (radial section) compared to the gouge layer (R = rotary side, S = stationary side); (b) SEM‐BSE image of the FIB sampling site; (c) orientation of FIB foils (Figure 6) and shear direction; and (d) geometric relationships among the commonly observed foliations in natural and experimentally deformed clay‐rich gouges (Passchier & Trouw, 2005). SEM‐BSE = scanning electron microscopy‐backscattered electron; FIB = focused ion beam.

After each experiment, the entire gouge layer was recovered from the sample holder. Water was removed from samples using a desiccator and then samples were embedded in epoxy resin. Then, after a curing time of at least 24 hr, the layer was sectioned along the radius (Figure 2a) and polished with an oil‐based suspension of diamond particles. We did not use water during sample preparation to avoid distortion of the microstructures due to smectite‐water interaction (expansion, shrinking, etc.). To obtain representative information on the deformation processes activated in the experiments, a field‐emission scanning electron microscope (JEOL JSM‐6500F, INGV, Rome, and FEI Quanta 650, University of Manchester) was used to investigate the radial sections and identify the volumes where gouge was deformed (Figures 4 and 5).

Electron‐transparent thin foils of the gouge layer were prepared with a FEI Helios Nanolab 3G FIB‐SEM. As all electron‐transparent FIB foils were cut orthogonal to the radial section, they were also subparallel to the local slip vector (Figures 2b and 2c). Sampling areas were within the deformed volumes characterized by the absence of clay‐opal grains found in the starting material (see Figures 4 and 5). Three FIB foils were sampled inside the deformed volumes of the gouge layers deformed at slip rates of 10^−5^ m/s for 0.1 m of slip and at 0.01, 0.1, and 1.3 m/s for 3 m of slip (see Figures 4 and 5). The position of sampling for the FIB foil was located as close as possible to the stationary side and to the outer radius of the gouge layers. Additionally, a FIB foil was sampled outside the deformed volume (i.e., ca. 500 μm toward the rotary side) of the gouge layer tested at 1.3 m/s for 3 m of slip (see Figures 4 and 5).

Electron‐transparent foils were investigated using (scanning) transmission electron microscopy ((S)TEM) in a FEI Talos F200X (S)TEM equipped with four energy‐dispersive X‐ray detectors (Super‐X EDX). All FIB‐SEM and TEM analyses were carried out at the Electron Microscopy Utrecht. Image analysis was performed on four dark field and bright field TEM images sampled both outside and inside the high‐strain domain (Figure S2). The length of smectite crystals was measured directly from the images. Binary images were produced by outlining the opal clasts manually and analyzed with Fiji software to obtain the equivalent diameter of the clasts (Figure S2 and Table S2).

## 3 Results

### 3.1 Mechanical Data and Modeled Temperatures

**Figure 3:**
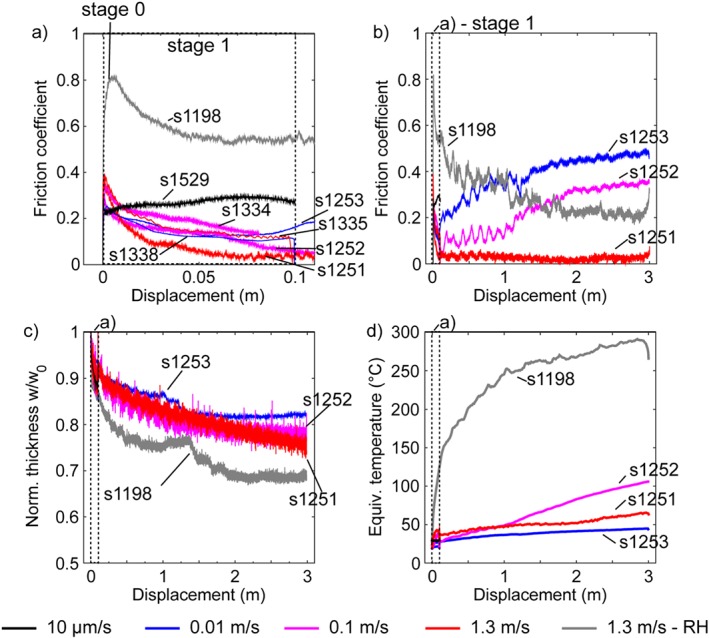
Experimental data. The evolution of friction coefficient with slip during (a) stage 0 and 1 (<0.08 m of slip) and (b) the entire experiment. Data corrected for the friction coefficient of the Teflon rings and sample assemblage (see section 2). (c) The evolution of normalized thickness with displacement. (d) Evolution of the equivalent temperature (from finite element method models) with slip (Figure 1b). Black boxes in panels (b)–(d) indicate the stage 1 represented in panel (a).

The apparent friction coefficient evolved with slip in three main stages during the partly saturated experiments (Figure 3). Stage 0 comprised the initial elastic and anelastic loading of the sample and experimental apparatus until the friction coefficient reached a peak value of *μ′* = 0.3–0.35. Stage 0 was followed by stage 1, lasting up to 0.08 m of slip, which included the decay of the friction coefficient to a minimum value (*μ′* = 0.05–0.15, Figure 3a). Instead, stage 2, lasting from 0.08 to 3 m of slip (Figure 3b), was slip rate dependent: the friction coefficient increased for *V* = 10^−5^, 0.01 and 0.1 m/s but remained constant and very low (*μ′* < 0.1, similar to Faulkner et al., 2011) for *V* = 1.3 m/s. The extrusion of gouge in all the experiments presented here was negligible; however, the gouge layer thickness decreased with displacement indicating compaction at all imposed slip rates and was accompanied by expulsion of water from the sample holder (Figure 3c). All the modeled maximum equivalent temperatures were very low in partly saturated experiments, usually lower than 50 °C and increased up to 103 °C only in the experiment performed at 0.1 m/s (Figure 3d and Table 2).

Comparing the experiment s1251 performed at a slip rate of 1.3 m/s under partly saturated conditions with the experiment s1198 performed at the same slip rate under room humidity conditions, we observe that (i) the initial peak friction increases from *μ′* = 0.4 (partly saturated) to *μ′* = 0.8 (under room humidity), (ii) the minimum friction coefficient increases from *μ′* = 0.05–0.1 to *μ′* = 0.2 (Figure 3c), (iii) the slip distance required to achieve the minimum friction increases from *d* < 0.1 m to *d* ~ 3 m (Figure 3c), and (iv) the maximum equivalent temperature was much lower in the partly saturated experiment (i.e., 65 °C), compared to the room humidity experiment (i.e., 300 °C, Figure 3d).

### 3.2 Microstructures

**Figure 4:**
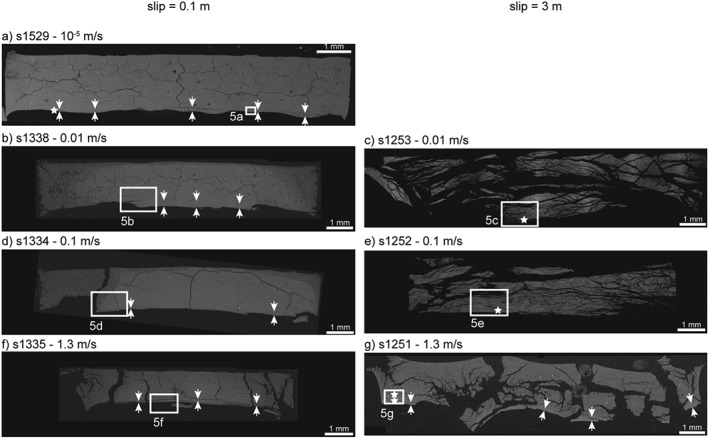
Backscattered electron (BSE)‐SEM images of the radial sections of the gouge layer, oriented as Figure 2a. Left column contains all the experiments stopped at 0.1 m of slip and right column those stopped at 3 m of slip. White arrows delimit the thickness of the deformed domain and white stars the FIB‐SEM sampling sites. Slip rate of the experiments are (a) 10^−5^ m/s, (b) and (c) 0.01 m/s, (d) and (e) 0.1 m/s, and (f) and (g) 1.3 m/s. White boxes: location of images (a) to (g) in Figure 5. SEM = scanning electron microscopy; FIB = focused ion beam.

**Figure 5:**
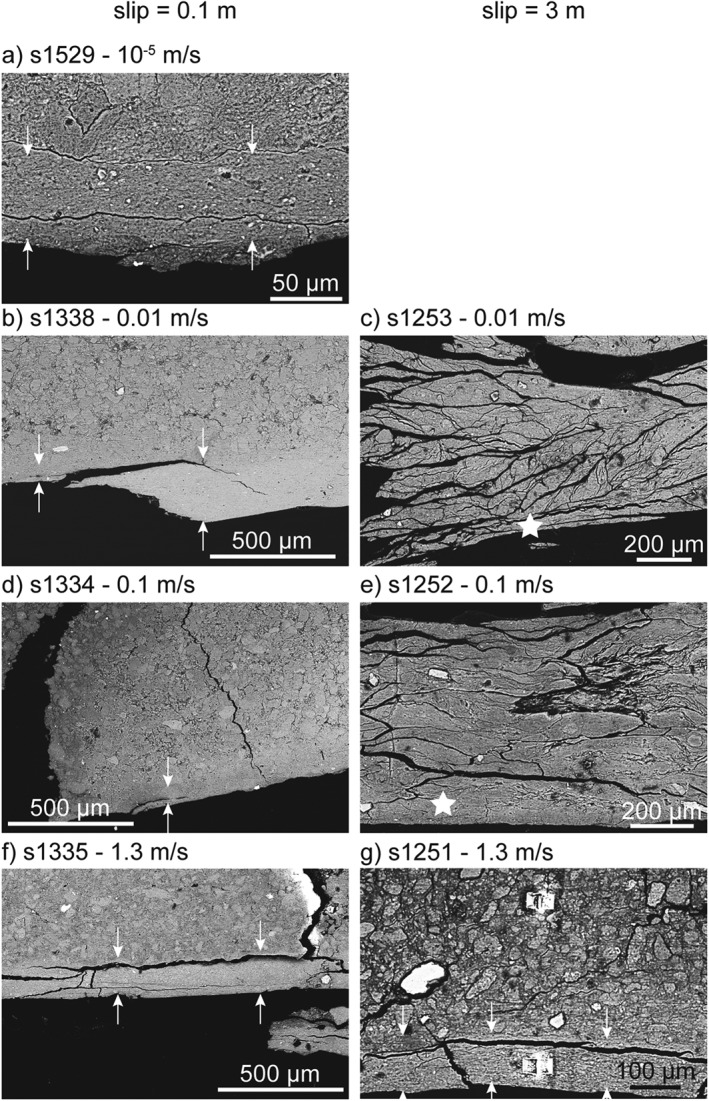
Backscattered electron (BSE)‐SEM images of the radial sections of the gouge layers oriented as Figure 2a, from the areas indicated in Figure 4. Left column contains all the experiments stopped at 0.1 m of slip and right column those stopped at 3 m of slip. White arrows delimit the thickness of the deformed domain and white stars the FIB‐SEM sampling sites. Slip rate of the experiments are (a) 10^−5^ m/s, (b) and (c) 0.01 m/s, (d) and (e) 0.1 m/s, and (f) and (g) 1.3 m/s. SEM = scanning electron microscopy; FIB = focused ion beam.

Microstructural observations conducted on the postexperiment radial sections under SEM showed that the granular texture of the starting material was locally obliterated (Figures 4 and 5). These volumes of the gouge layer in which the granular texture of the starting material was obliterated were recognized and their average thickness was measured from the backscattered electron (BSE‐)SEM images (Figures 4 and 5). The ratio of the slip rate of the experiment and the measured thickness defined the maximum estimate of the shear strain rate achievable during the experiment, which depended on the combination of slip rate and displacement (Table 2 and Figures 4 and 5). In all the partly saturated experiments that stopped at 0.1 m of slip, independently of the imposed slip rate, deformation localized in a <300‐μm‐thick volume, showing sharper boundaries at 0.01–1.3 m/s compared to 10^−5^ m/s. (i.e., cf. Figure 5a with Figures 5b, 5d, and 5f). In the experiments that stopped after 3 m of slip, deformation localized in a ~150‐μm‐thick volume only when the gouge layer was sheared at *V* = 1.3 m/s and, instead, the whole gouge layer appeared deformed in the experiments performed at *V* = 0.01 and 0.1 m/s (cf. Figure 5g with Figures 5c and 5e).

**Figure 6:**
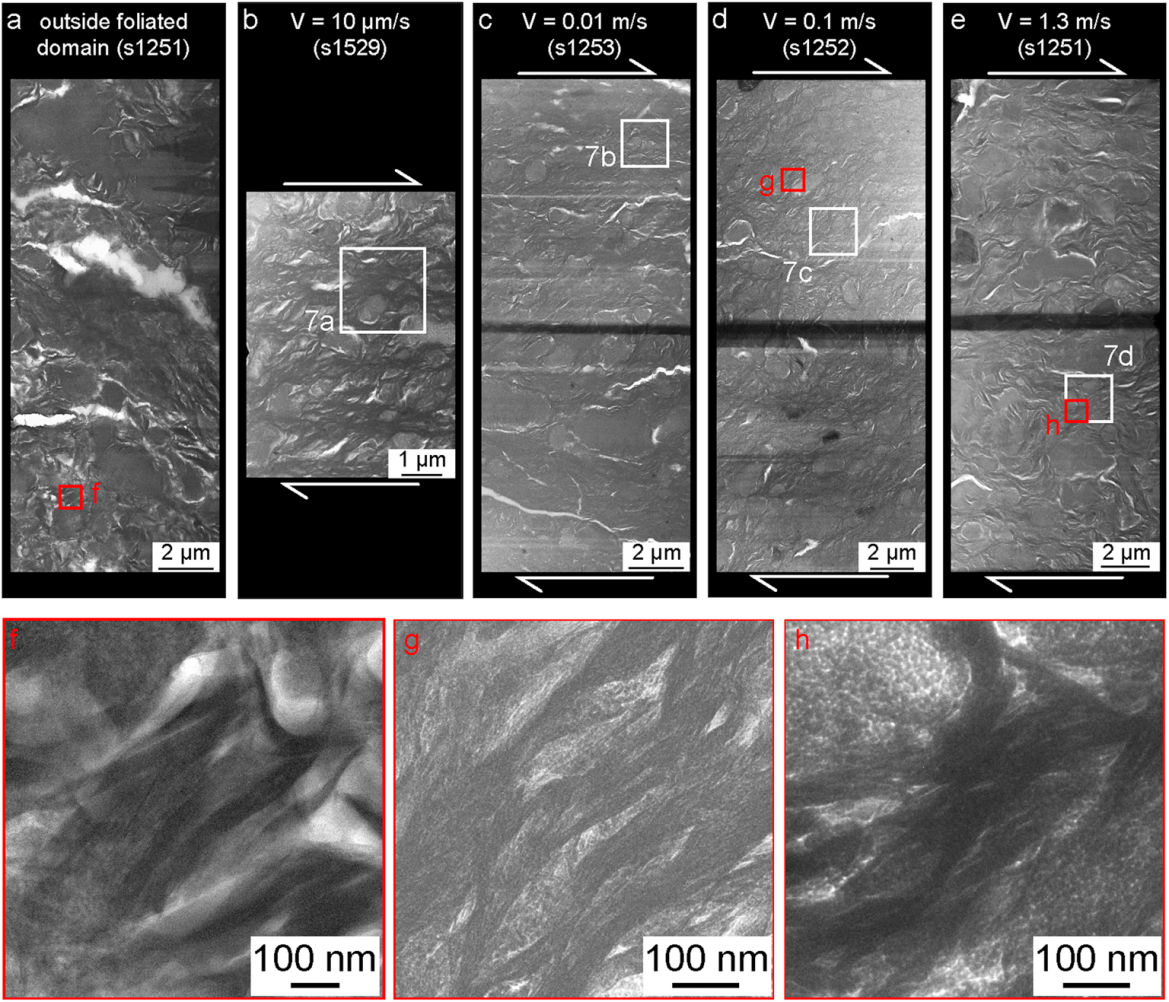
Nanoscale imaging of the FIB‐SEM foils sampled outside and inside the deformed domain. Bright field (BF)‐STEM images of the FIB‐SEM foils sampled (a) outside the deformed domain (experiment s1251); inside the deformed domain in the experiments performed at *V* = 10^−5^ m/s (0.1 m of slip: s1529, panel b) and *V* = 0.01, 0.1, and 1.3 m/s (3 m of slip: s1253, s1252, and s1251, panels c to e). Red boxes show the position of the nano‐images to the bottom of the figure (panels f to h). White boxes: location of nano‐images a to d in Fig. 7. FIB‐SEM = focused ion beam scanning electron microscopy; STEM = scanning transmission electron microscope.

**Figure 7:**
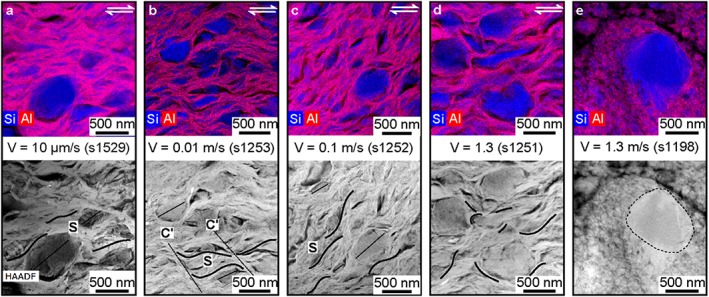
Nanoscale imaging of the nanofoliation in the partially saturated foliated domain compared with a nanoparticle‐rich domain from an experiment conducted under room humidity conditions (s1198 from Aretusini et al. (2017)). TEM compositional maps, with Al and Si EDX intensities combined in red and blue colors, respectively (top) and high‐angle annular dark field (HAADF)‐STEM images (bottom). Panels (a) to (d) show the nanofoliation developed at all slip rates under wet conditions (white boxes in Figure 6). Panel (e) shows the nanoparticle‐rich domain developed at slip rate of 1.3 m/s (equivalent to experiment s1251) but performed under room humidity conditions (Aretusini et al., 2017). TEM = transmission electron microscopy; EDX = energy‐dispersive X‐ray detectors; STEM = scanning transmission electron microscope.

The nanoscale investigation was conducted both within and outside the deformed gouge volumes (Figures 4 and 5). Independently of the imposed slip rate, all deformed volumes contained a “nanofoliation”: a foliation made of aligned subparallel smectite crystals anastomosing around opal clasts which was observable only at the nanoscale (Figures 6 and 7). The shape of ~10‐nm‐thick elongated smectite crystals was approximately tabular outside the foliated domain (Figures 6a and 6f) and sigmoidal within it (Figures 6b–6d, 6g, 6h, and 7a–7d). Opal crystals were dominantly angular in the foliated domain (Figures 6b–6d and 7a–7c). Opal grain size decreased in size in the foliated domain (~180 nm) compared to the volumes outside it (~ 400 nm), as evidenced by the image analysis of TEM images (Table S2 and Figure S3). Image analysis showed that at all slip rates the long axes of smectite and of opal grains were mainly aligned at 30° to 50° to the horizontal axis of the image (Figure S3).

The comparison of the microstructures produced in the experiments performed at 1.3 m/s under partly saturated versus room humidity conditions shows differences (i) in the thickness of the deformed domain (150–300 μm vs. 1,000 μm, respectively), but especially (ii) in the nanoscale fabric within the domain (Figure 7). While in the experiments performed under room humidity conditions the deformed gouge volumes are mainly made by subrounded smectite nanoparticles and smectite crystals wrapping opal fragments (Figure 7e, clay cortex aggregates; Aretusini et al., 2017; Boutareaud et al., 2008; Ferri et al., 2011), in the experiments conducted in partly saturated conditions, intact smectite crystals define the nanofoliation (Figure 7d).

## 4 Discussion

### 4.1 Microstructures and Proposed Deformation Processes

**Figure 8:**
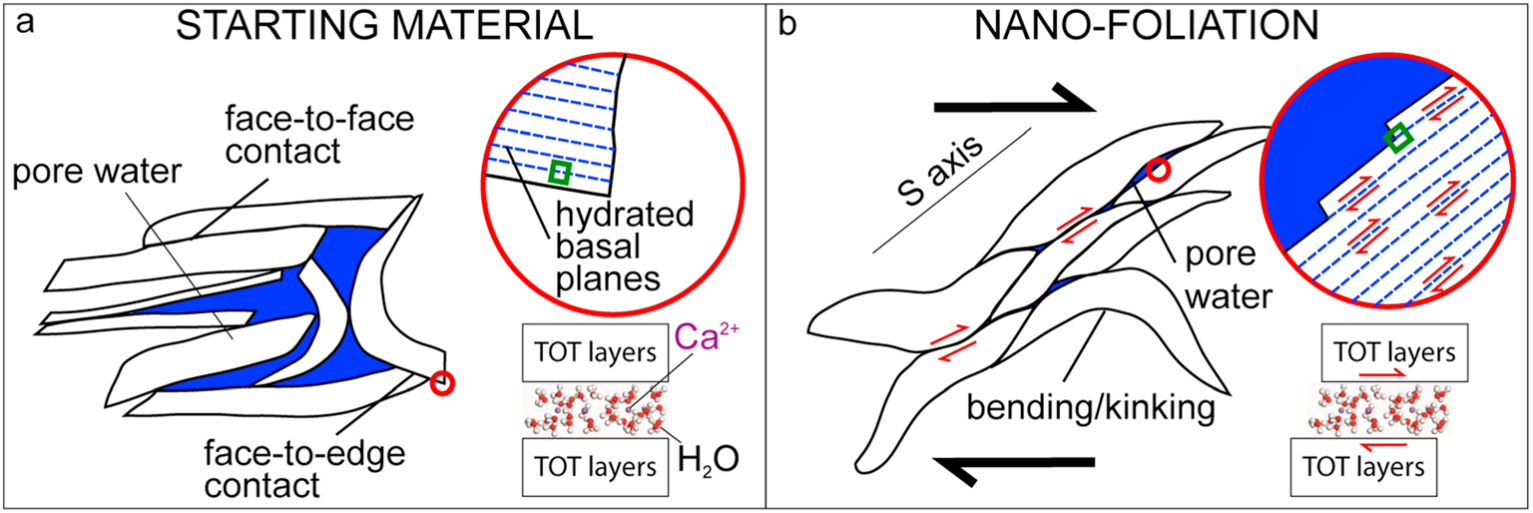
Sketch summarizing the deformation processes proposed to be active at subseismic to seismic slip rates in the nanofoliation within the foliated domain. (a) Areas outside the foliated domain are compared to (b) the nanofoliation within the foliated domain.

The nanoscale images showed that both smectite crystals and opal clasts had first‐order changes in geometrical disposition, in shape, and grain size comparing foliated to nonfoliated areas (Figures 6 and 7). Nanofoliations were characterized by a higher alignment of clays (i.e., mainly parallel to S direction, see Figure S3), with predominant sigmoidal shapes (e.g., cf. Figure 6a with Figures 6b–6e) and smaller grain sizes (reduced by ~1/2, Figure S3) than the volumes outside it. We suggest that hydrated grain boundaries were lubricated and facilitated the alignment and rotation of clays during shear deformation. We also suggest that water molecules lubricated the basal plains, which were guiding the change in shape of clays (possibly by relative displacement of TOT layers along basal planes, Figure 8) and the grain size reduction by delamination along the basal planes themselves. Similarly as clays, opal clasts in the nanofoliation showed alignment with the S direction (e.g., in Figures 6b and 6c) and smaller grain sizes (reduced by ~1/2 to 3/4, see Figure S3), compared to the nonfoliated volumes. The widespread presence of angular opal clasts in the nanofoliation implied that cataclasis by clast interaction and indentation was the main deformation mechanism operating in opal at all shear strain rates. Other processes, as diffusive mass transfer, are excluded, because in our experiments we did not recognize any overgrowth texture indicating reprecipitation of opal. Moreover, the shear strain rates predicted by diffusive mass transfer equations were much lower than those measured from the SEM images (Text S2, using methodology as described in Den Hartog & Spiers, 2014; Rimstidt & Barnes, 1980; Tembe et al., 2010; Tester et al., 1994).

The arrangement of smectite and elongated opal crystals had second‐order changes from low to high strain rates (Figures 6 and 7). The alignment of elongated opals and clays appeared stronger at low compared to high maximum strain rates from the qualitative observation of TEM lamellae (e.g., cf. Figure 6c with Figure 6e). However, quantitative image analysis of TEM micrographs (Figure S2) showed that opal and clay grains equally align parallel to a dominant angle of 30–50°, parallel to the S direction (Figure S3). Possibly, image analysis captured local characteristics of the nanofoliation. On the other hand, the interaction between opal and smectite crystals could influence the degree of alignment (i.e., the intensity of fabric) in the nanofoliation: areas with smaller opal clasts grain size also appeared to have the highest degree of alignment within the same TEM lamella (see Figure 6e).

Remarkably, the same smectite‐rich gouges sheared under identical deformation conditions (slip rate, normal stress, displacement), but in the absence of liquid water (i.e., room humidity conditions) developed rounded nanoparticles from comminution of the smectite crystals and opal grains and the nanofoliation was absent (Aretusini et al., 2017) (cf. Figures 7d and 7e). The presence of water along basal planes and grain boundaries and in pore spaces triggered different deformation mechanisms, which determined the resulting frictional strength of the sheared gouge (Bullock et al., 2015). In the experiment performed at *V* = 1.3 m/s (s1251), the presence of water‐lubricated smectite surfaces resulted in the formation of the nanofoliation and contributed to the achievement of an extremely low friction coefficient after few centimeters of slip (Figures 3a and 3b).

### 4.2 The Apparent Friction Coefficient of the Nanofoliation

Technical challenges related to gouge confinement and pore pressure control in rotary shear machines worldwide limit a comprehensive understanding of whether the measured shear stress (i.e., *τ=μ(σ‐Pf)*) evolution with slip in experiments performed at seismic slip rates on clay‐rich gouges depends on variations of the friction coefficient (i.e., *μ*) or of the fluid pressure (i.e., *Pf*). In fact, frictional weakening could be likewise related to the pore fluid pressure transient increase and to the decrease of the friction by increase of the thickness of water films on smectite basal planes (Moore & Lockner, 2007) and grain boundaries. In our experiments, like in those performed with other rotary shear machines (Faulkner et al., 2011; Ferri et al., 2011; Remitti et al., 2015; Ujiie et al., 2013), pore fluid pressure was not controlled nor monitored and therefore full saturation was not achieved before the experiment. The resulting friction coefficient is rather an apparent friction coefficient (i.e., *μ′=τ/σ*, Figure 3) which can be either higher (because of partial saturation) or lower (because it is not corrected for the effect of pore pressure) than the friction coefficient (i.e., *μ=τ/(σ‐Pf)*). Moreover, due to the partial sealing of the gouge layer exerted by the Teflon ring, water can be expelled during shear deformation favoring the fluid drainage from the slipping zone. In addition, smectite‐rich gouges have a low hydraulic diffusivity of 2.3·10^−8^ ≤ *κ* ≤ 10^−7^ m^2^/s (typical values for smectite‐rich natural fault gouges,Faulkner et al., 2018 ; Wibberley, 2002), therefore the equilibration of the transient pore pressures due to pore volume decrease by shear compaction cannot occur during shear deformation at shear strain rates of ~6.6·10^−4^ s^−1^ (calculated using slip rates of 0.5 μm/s and a gouge layer thickness of ~0.75 mm, Faulkner et al., 2018). At seismic slip rates, equilibration is further complicated as the pore pressure increase by shear compaction sums with the transient pore pressure increase induced by thermal pressurization of the pore fluids (Faulkner et al., 2011), by thermochemical pressurization resulting from the expulsion of water films from the basal planes (completed at *T* > 120–150 °C, Ferri et al., 2011), or by the vaporization of pore water (Chen et al., 2017). The combination of the low hydraulic diffusivity of smectite‐rich gouges with thermal pressurization effects implies that the mechanical behavior cannot be explained in a model without accurately measuring at least pressure, temperature, and porosity change. Dilatancy hardening might occur as well at all slip rates, during shear in strain localized foliations and reduce the pressurization of pore fluid. However, our gouge thickness measurements seem to suggest that this effect is either significantly lower than our detection limit or outpaced by the shear‐enhanced compaction.

During our experiments under partly saturated conditions, the evolution of the apparent friction coefficient varied with slip and slip rate (see the long strengthening stage 2 for *V* = 0.01–0.1 m/s, Figure 3). This evolution is possibly related to the characteristic time for diffusion of water *t*_*c*_ *= d* ^*2*^ */κ*, where *d* is half the gouge layer thickness and *κ* is hydraulic diffusivity (Faulkner et al., 2018; Wibberley, 2002). Assuming a minimum hydraulic diffusivity of 10^−8^ m^2^/s (same order of magnitude of smectite‐rich gouges, Faulkner et al., 2018), *t*_*c*_ ranged from 0.46 to 2.25 s depending on the thickness of the foliated domain during stage 1 (i.e., 150–300 μm). The range of values for *t*_*c*_ are of the same magnitude of the time duration of stage 1 (i.e., 0.08 to 10 s, Table 2). This analysis suggests that the clay‐rich layer that evolved into the high strain domain was not fully drained at the initiation of slip and pressurization due to shear‐enhanced compaction plus thermal pressurization (possibly for *V* ≥ 0.01 m/s) weakened the experimental fault by decreasing the effective normal stress. This initial short‐lasting undrained state was enhanced by the impermeable nature of the steel sample holder (similar to the effect of using impermeable bounding rocks in Faulkner et al., 2011, and Ujiie et al., 2013). With increasing slip and for *t* > *t*_*c*_, the diffusion of pore water outside of the foliated domain and its expulsion along the Teflon‐sample holder interface (Boundary 2 in Figure 1b), resulted in the dissipation of the pore pressures developed in the gouge layer during stage 1 and, consequently, in the observed slip strengthening behavior (stage 2, Figure 1b). However, since pore fluid pressure was not monitored adjacent to the slipping zone, we cannot exclude that the apparent friction coefficient increased during slip strengthening stage 2 by a simultaneous decrease in pore fluid pressure and increase of the friction coefficient. The increase of the friction coefficient could be related to the rearrangement of clay particles resulting in densification of the nanofoliation. Moreover, the friction coefficient should increase with the decrease of the thickness of water films in smectite basal planes (Moore & Lockner, 2007). As a consequence, the measured increase of the friction coefficient could be related to the progressive expulsion of water from the sample, and in shearing of the nanofoliation under partially saturated conditions (Morrow et al., 2017).

### 4.3 Nanofoliation as a Marker of Slow and Fast Fault Slip

Natural clay‐rich fault rocks are often foliated, from the tens of meters scale, displaying “scaly clays” fabric (Vannucchi et al., 2003, and references therein) in subduction zones faults (Chester et al., 2013; Kirkpatrick et al., 2015) and in landslide decollements (Larue & Hudleston, 1987). But natural clay‐rich fault rocks are also foliated up to the micrometer scale within principal slip zones in fault gouges (e.g., Rutter et al., 1986). The occurrence of a foliated fabric at the microscale was interpreted as the result of the crystallization of new clay minerals (Schleicher et al., 2010), also combined with cataclasis, grain size reduction and grain rotation (Rutter et al., 1986). The development of such foliations is normally considered related to aseismic creep, consistently with the velocity strengthening behavior of smectite‐rich gouges (Morrow et al., 2017, and references therein).

Based on the experimental and nanostructural evidence reported here, we suggest that there are no first‐order microstructural differences between subseismic and seismic foliations. This similarity between slow to fast foliations applies to natural principal slip zones (i.e., where strain‐localization occurred) and limitedly to the microscale to the nanoscale foliations formed by the geometrical rearrangement of clay particles. In fact, our experiments could not reproduce the crystallization of new clay minerals during deformation at subseismic slip rates. This similarity in the foliations across slip rates could be explained by the action of the same deformation process: frictional slip in smectite along basal planes (i.e., delamination) and grain boundaries in the presence of a water‐lubricating film which together promote grain size reduction, alignment, and rotation of the clays. In particular, delamination was shown to be active at strain rates ranging from 5·10^−9^ s^−1^ to 10^−6^ s^−1^ (as reported by French et al., 2015) but also, as proposed here, at strain rates up to ~10^4^ s^−1^. As similar deformation mechanisms are active over a broad range of strain rates, at the shallow crustal depths at which smectite clays are stable, both aseismic creep and seismic ruptures propagating from depth can result in the formation of nanosale to microscale foliations.

The distinction between fabrics produced at subseismic versus seismic strain rates could rely on the thickness of the foliated domains. A very low thickness (i.e., below ~150 μm), indicated high strain localization, which is a characteristic of natural principal slip zones undergoing seismic slip (Rice, 2006). However, we showed that the thickness was largely dependent on displacement. In the short experiments (0.1 m) thin foliated domains occurred at all slip rates, but strain localization was more evident at 0.01, 0.1, and 1.3 m/s (Figure 5a vs. Figures 5b, 5d, and 5f). In the long experiments (3 m) strain localization was evident only at 1.3 m/s (Figure 5g). Therefore, strain localization was typical of all seismic slip rates but was exclusive of coseismic slip rates (>1 m/s) only with large displacements.

Rather than the thickness of the nanofoliation fabric, perhaps a more robust marker of seismic slip in smectite‐rich gouges would be the presence of microstructural and mineralogical evidences of abrupt thermal pulses that can indicate the high power dissipation (i.e., the product of shear stress and slip rate) during natural earthquakes (for a summary, see Rowe & Griffith, 2015). These evidences could be smectite dehydration and transition from smectite to illite‐like structure (Ferri et al., 2011) or to chlorite (Kameda et al., 2011). Other evidences of the thermal pulses during past earthquakes could be recorded both in the foliation and in the accessory minerals, as thermal breakdown (Collettini et al., 2013) or just in accessory minerals, in the degree of crystallization of amorphous carbon (Kuo et al., 2017), or of vitrinite (Sakaguchi et al., 2011), or in the thermal maturity of organic matter compounds (Savage et al., 2018).

## 5 Conclusions

We suggest that frictional slip along water‐lubricated smectite grain boundaries and basal planes occurs from subseismic to seismic slip rates in principal slip zones of natural smectite‐rich faults found in subduction zones at shallow depth (e.g., in the Japan Trench), or in large landslide decollements (e.g., the 1963 Vajont landslide). As frictional slip along water‐lubricated smectite grain boundaries and basal planes was proposed to be active over a wide range of shear strain rates (6 orders of magnitude in the experiments presented here), nanofoliations can develop from the subseismic to the coseismic stages if water is available along the smectite grain boundaries and basal planes. At the microsale and nanoscale, foliated smectite‐rich fault gouges can be produced either during subseismic or seismic slip. However, a highly localized foliation could be a marker of seismic slip rates (*V* ≥ 0.01 m/s) when recognized in natural fault rocks.

## Supporting information

Supporting Information S1
