# Supplementary material for: Subseismic to Seismic Slip in Smectite Clay Nanofoliation
Source: J Geophys Res Solid Earth. 2019 Jul 29;124(7):6589–601. doi: 10.1029/2019JB017364 (PMC6919425; doi:10.1029/2019JB017364)
Supplement: Supplementary file 1 — Supporting Information S1 [file JGRB-124-6589-s001.docx]

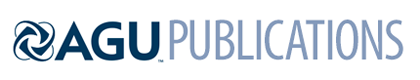


*Journal of Geophysical Research*

Supporting Information for

**Sub-seismic to seismic slip in smectite nano-foliation**

S. Aretusini^1^, O. Plümper^2^, E. Spagnuolo^1^, and G. Di Toro^1,3^

1. Sezione di Tettonofisica e Sismologia, Istituto Nazionale di Geofisica e Vulcanologia, Roma, Italy.

2. Department of Earth Sciences, Utrecht University, Utrecht, The Netherlands.

3. Dipartimento di Geoscienze, Università degli Studi di Padova, Padova, Italy.

**Contents of this file**

Text S1 to S2

Figures S1 to S3

Tables S1 to S2

**Introduction**

This supporting information contains:

1) The description and the results of the grain size analysis of the starting material tested in the high velocity friction experiments (Text S1, Figure S1, and Table S1).

2) The results of the image analysis on the scanning transmission electron microscope (STEM) images (Figure S2 and Table S2).

3) The calculation of compaction and strain rates by diffusive mass transfer (Text S2).

Text S1.

We dispersed 0.1 g of starting material STx-1b in a Na_3_PO_4_ aqueous solution (concentration of 2 g / 100 mL) to avoid flocculation of clay particles. The particle size distribution of the starting material was measured with a LUMiReader analytic instrument (Detloff et al., 2011) which illuminated the suspension and measured the space and time resolved extinction profiles for three incident wavelength (470, 630, and 870 nm in Figure S1). Reduced extinction profiles were correlated to the clarification of the suspension over time due to the sedimentation of particles. The volume weighted particle size distribution was calculated from the extinction profiles
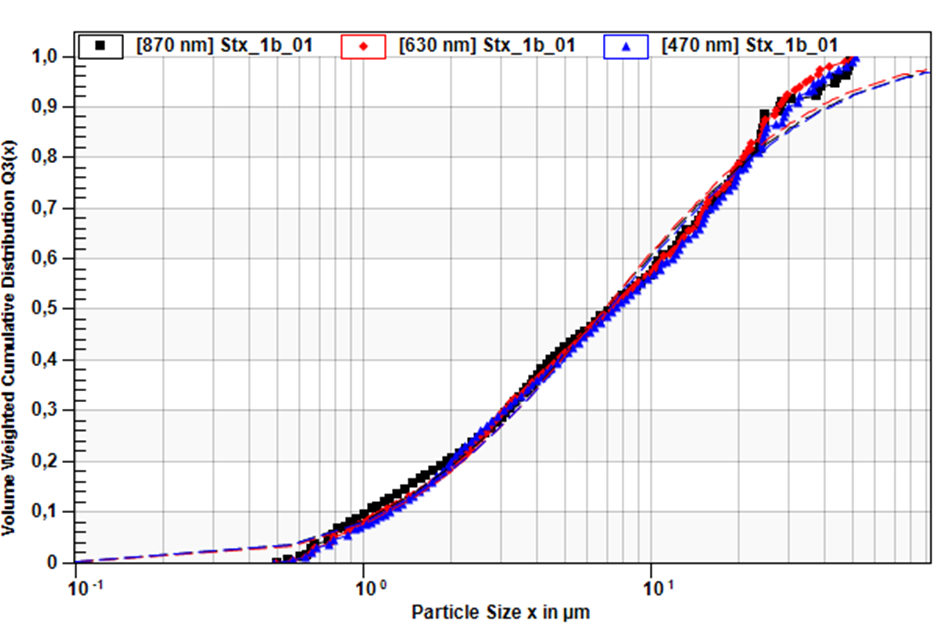
(dashed lines in Figure S1) and reported in Table S2.

Figure S1. Volume weighted particle size distribution of the starting material STx-1b. The volume weighted cumulative distribution (dots) was calculated from the extinction profiles and then fitted (dashed lines).


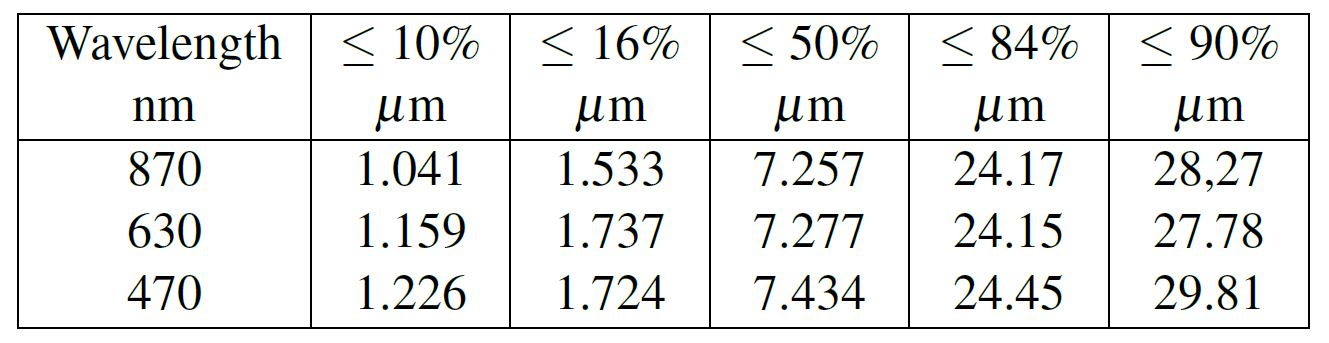


Table S1. Volume weighted particle size distribution of the starting material STx-1b.


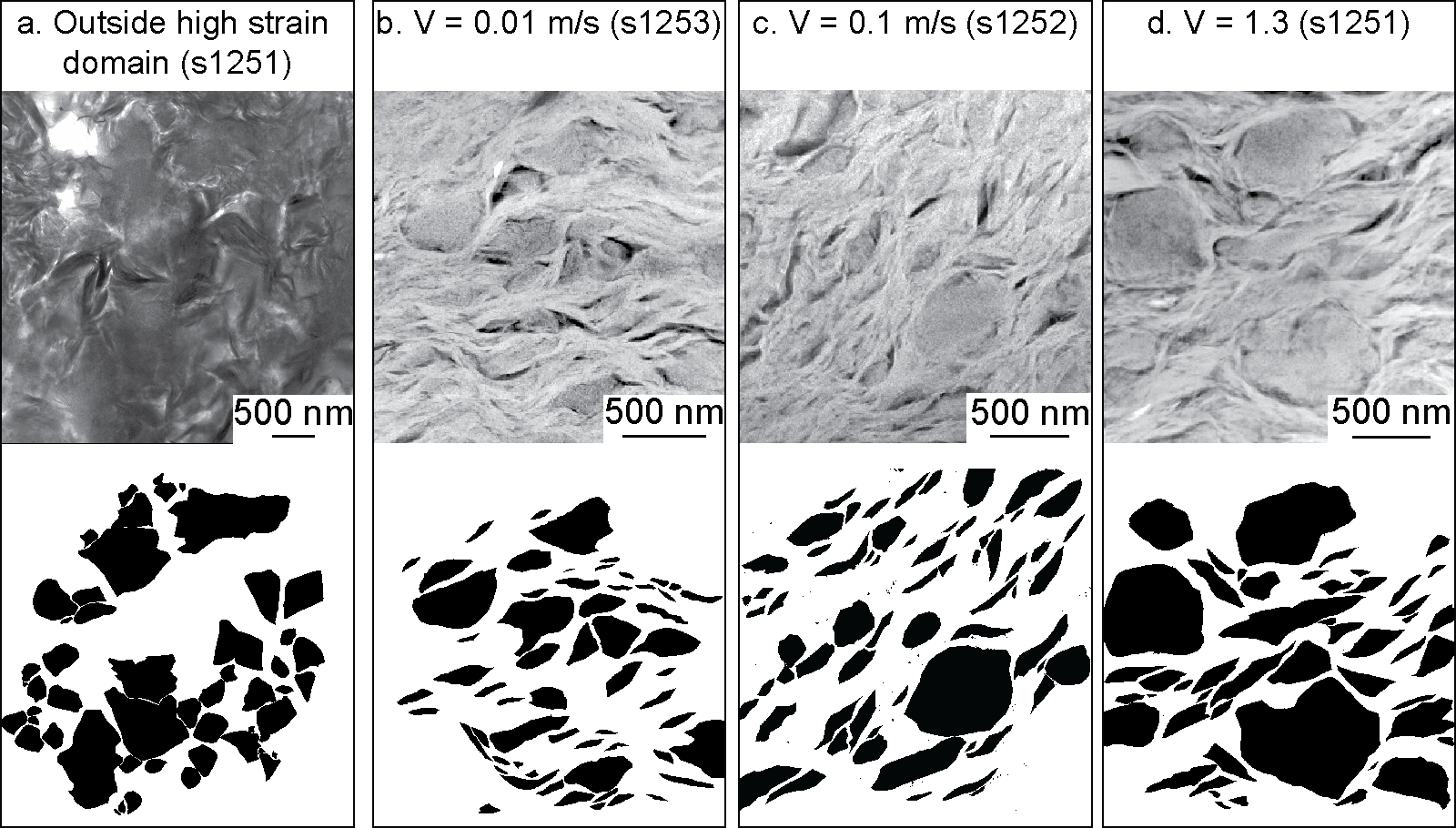


Figure S2. Scanning transmission electron microscope (STEM) images (Fig. 5 of main text) and binary images of opal clasts. a) is imaged from sample s1251, outside of the high-strain domain; b) to d) are imaged within the high strain domain from samples s1253 (*V* = 0.01 m/s), s1252 (*V* = 0.1 m/s), and s1251 (*V* = 1.3 m/a), respectively.


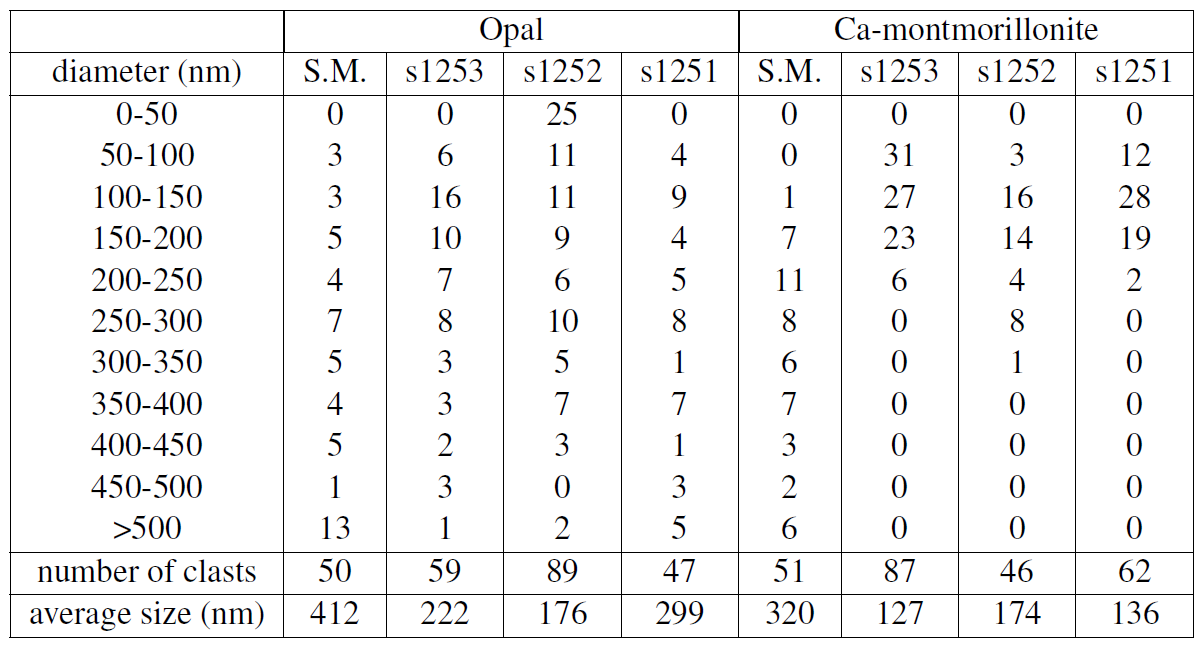
Table S2. Feret diameter calculated with Fiji for opal clasts and measured length of Ca-montmorillonite crystals. Diameter was extracted from images both outside (S.M., experiment s1251) and inside the high-strain domain (experiments s1253, s1252, and s1251 were performed at *V* = 0.01, 0.1 and 1.3 m/s).


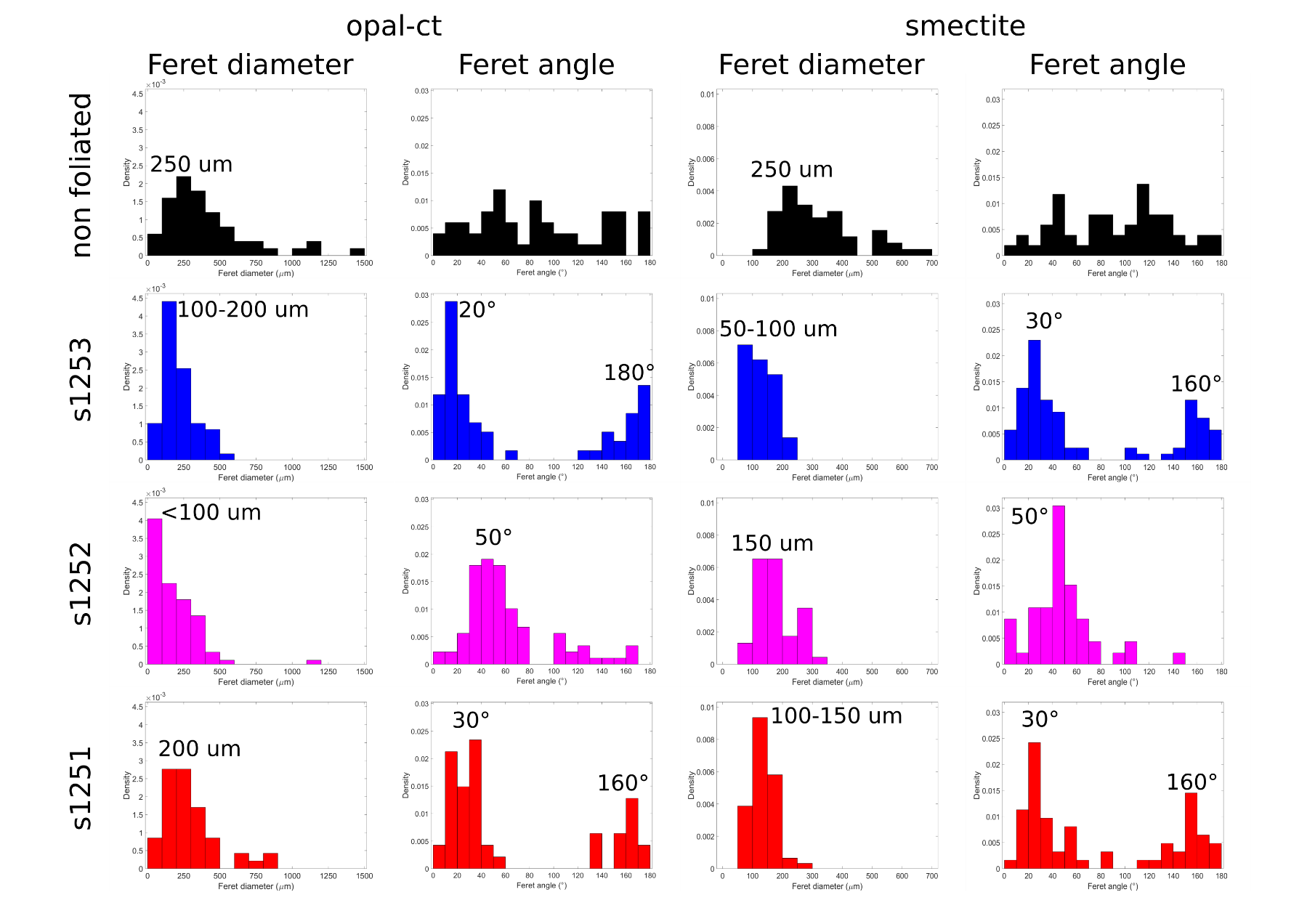


Figure S3. Results of image analysis. Feret diameter, or the maximum diameter of the clast, and Feret angle, the angle between the Feret diameter direction and the x-axis of the STEM images. Feret diameter and angle were extracted from images both outside (S.M., experiment s1251) and inside the high-strain domain (experiments s1253, s1252, and s1251 were performed at *V* = 0.01, 0.1 and 1.3 m/s).

Text S2.

The nano-foliation developed in the rotary shear experiments presented in the main text appeared similar to the illite-quartz system studied at sub-seismic slip rates (10^-8^ to 10^-3^ m/s), in which shear strain rates and compaction rates were controlled by the slowest process between deformation of quartz by thermally activated diffusive mass transfer and dilatant frictional slip on the illite foliation (Den Hartog & Spiers, 2014). We evaluated the shear strain and compaction strain rate by diffusive mass transfer in opal comparing the dissolution-precipitation kinetic parameters of quartz and amorphous silica. Shear strain rate accommodated by diffusive mass transfer in microstructural volumes in which shear stress is supported by individual ($\dot{\gamma_{qtz-b}}$) and overlapping ($\dot{\gamma_{qtz-o}}$) opal clasts, and compaction strain rate accommodated by pressure solution ($\dot{\epsilon_{\mathrm{comp}}}$) were calculated as (Den Hartog & Spiers, 2014):

$\dot{\gamma_{qtz-b}}=\frac{\pi k\tau\Omega^{2}}{\mathrm{RT}}\frac{D-2x}{D\left( D-x \right)}$ Eq. S1

$\dot{\gamma_{qtz-o}}=\frac{\pi k\tau\Omega^{2}}{\mathrm{RT}}\frac{1}{\sqrt{Dx-x^{2}}}$ Eq. S2

$\dot{\epsilon_{\mathrm{comp}}}=\frac{2k\sigma_{n}^{\mathrm{eff}}\Omega^{2}}{\mathrm{RT}}\frac{A}{\left( D-x \right)\mathrm{DL}}$ Eq. S3

In Eqs. S1 to S3, *k* is the reaction rate for dissolution-precipitation calculated at *T*, the maximum temperature achieved in our experiments (103 °C) for quartz (2.27 10^-11^ mol m^-2^ s^-1^, Rimstidt & Barnes, 1980) or for amorphous silica (1.56 10^-10^ mol m^-2^ s^-1^, Rimstidt & Barnes, 1980), *Ω* the molar volume of quartz (2.27 10^-5^ m^3^mol^-1^), *R* the Boltzmann constant, *τ* the shear strength of quartz (i.e., 0.7 σ_n_, Tembe et al., 2010), *σ_n_^eff^* the effective normal stress (i.e., 5 MPa, from our experiments). The other microstructural variables in Eqs. S1 to S3 were: *D* the clast diameter, *x* the vertical overlap length between clasts, *A* the pore area (calculated considering a porosity of 35%, see Den Hartog & Spiers, (2014)), and *L* the horizontal spacing of clasts. Among the microstructural variables, only *D* and *x* were independent variables. The particle diameter *D* and the clast overlap *x* were determined from TEM images (180 nm, average of 195 clasts, and 80 nm, respectively, see Tab. S2). Based on Eqs. S1-S3, we estimate that diffusive mass transfer can accommodate, with amorphous silica dissolution kinetics, a maximum shear strain rate of 10^-9^ s^-1^ in overlapping areas between clasts and of 10^-10^ s^-1^ within each clast, and compaction strain rates of 10^-9^ s^-1^. Such estimated strain rates changed to 10^-10^, 10^-11^, and 10^-10^ s^-1^, respectively, considering quartz dissolution kinetics. All strain rate values are orders of magnitude lower than the minimum strain rate (i.e., 10^-2^-10^4^ s^-1^) achieved in the experiments presented here and where nano-foliations were produced.

**References**

Den Hartog, S. A. M., & Spiers, C. J. (2014). A microphysical model for fault gouge friction applied to subduction megathrusts. *Journal of Geophysical Research: Solid Earth*, *119*(2), 1510–1529. https://doi.org/10.1002/2013JB010580

Detloff, T., Sobisch, T., Lerche, D., GmbH, L., & Straße, J.-L. (2011). Characterisation of separating dispersions by multi wavelength extinction profiles, 4.

Rimstidt, J. D., & Barnes, H. L. (1980). The kinetics of silica-water reactions. *Geochimica et Cosmochimica Acta*, *44*(11), 1683–1699. https://doi.org/10.1016/0016-7037(80)90220-3

Tembe, S., Lockner, D. A., & Wong, T.-F. (2010). Effect of clay content and mineralogy on frictional sliding behavior of simulated gouges: Binary and ternary mixtures of quartz, illite, and montmorillonite. *Journal of Geophysical Research*, *115*(B3). https://doi.org/10.1029/2009JB006383

Tester, J. W., Worley, W. G., Robinson, B. A., Grigsby, C. O., & Feerer, J. L. (1994). Correlating quartz dissolution kinetics in pure water from 25 to 625°C. *Geochimica et Cosmochimica Acta*, *58*(11), 2407–2420. https://doi.org/10.1016/0016-7037(94)90020-5
